# Novel Papaverine Metal Complexes with Potential Anticancer Activities

**DOI:** 10.3390/molecules25225447

**Published:** 2020-11-20

**Authors:** Ahmed Gaber, Walaa F. Alsanie, Deo Nandan Kumar, Moamen S. Refat, Essa M. Saied

**Affiliations:** 1 Department of Biology, College of Science, Taif University, P.O. Box 11099, Taif 21944, Saudi Arabia; a.gaber@tu.edu.sa; 2 Department of Clinical Laboratories, College of Applied Medical Sciences, P.O. Box 11099, Taif 21944, Saudi Arabia; w.alsanie@tu.edu.sa; 3 Department of Chemistry, Deshbandhu College, University of Delhi, Delhi 110019, India; dnandan2002@gmail.com; 4 Department of Chemistry, College of Science, Taif University, P.O. Box 11099, Taif 21944, Saudi Arabia; 5 Department of Chemistry, Faculty of Science, Port Said University, Port Said 42511, Egypt; 6 Chemistry Department, Faculty of Science, Suez Canal University, Ismailia 41522, Egypt; 7 Institute for Chemistry, Humboldt Universität zu Berlin, Brook-Taylor-Str. 2, 12489 Berlin, Germany

**Keywords:** papaverine, bioactive metal complex, X-ray structure, nanostructure, antibacterial, anticancer, pharmacological application

## Abstract

Cancer is one of the leading causes of death worldwide. Although several potential therapeutic agents have been developed to efficiently treat cancer, some side effects can occur simultaneously. Papaverine, a non-narcotic opium alkaloid, is a potential anticancer drug that showed selective antitumor activity in various tumor cells. Recent studies have demonstrated that metal complexes improve the biological activity of the parent bioactive ligands. Based on those facts, herein we describe the synthesis of novel papaverine–vanadium(III), ruthenium(III) and gold(III) metal complexes aiming at enhancing the biological activity of papaverine drug. The structures of the synthesized complexes were characterized by various spectroscopic methods (IR, UV–Vis, NMR, TGA, XRD, SEM). The anticancer activity of synthesized metal complexes was evaluated in vitro against two types of cancer cell lines: human breast cancer MCF-7 cells and hepatocellular carcinoma HepG-2 cells. The results revealed that papaverine-Au(III) complex, among the synthesized complexes, possess potential antimicrobial and anticancer activities. Interestingly, the anticancer activity of papaverine–Au(III) complex against the examined cancer cell lines was higher than that of the papaverine alone, which indicates that Au-metal complexation improved the anticancer activity of the parent drug. Additionally, the Au complex showed anticancer activity against the breast cancer MCF-7 cells better than that of cisplatin. The biocompatibility experiments showed that Au complex is less toxic than the papaverine drug alone with IC_50_ ≈ 111µg/mL. These results indicate that papaverine–Au(III) complex is a promising anticancer complex-drug which would make it a suitable candidate for further in vivo investigations.

## 1. Introduction

**Figure 1:**
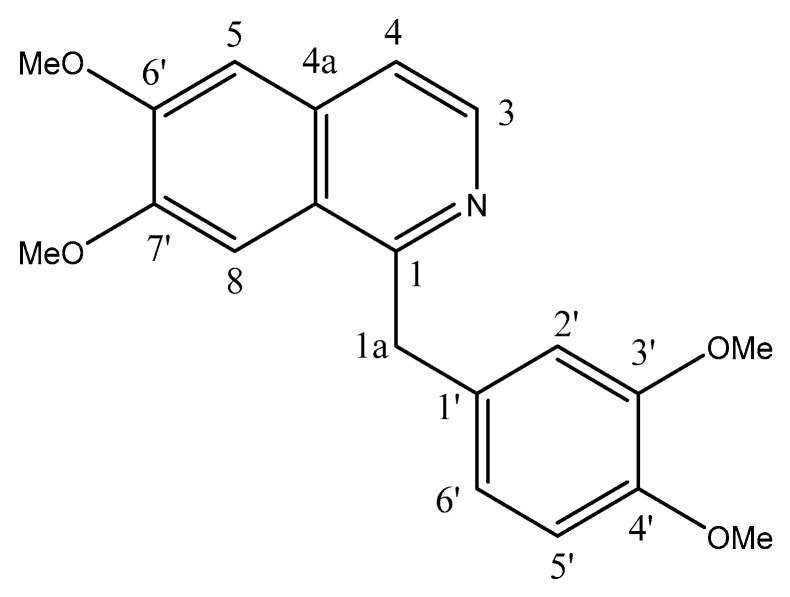
Chemical structure of papaverine drug.

With over 9.6 million deaths in 2018, cancer is the second leading cause of death worldwide [1]. Therefore, there is an urgent demand to develop novel anticancer drugs with high bioactivities and non-conventional modes of action [2,3,4]. Papaverine is an opiate alkaloid isolated from *Papaver somniferum* and *Rauwolfia serpentina* plants. Chemically, papaverine (1-[(3,4-dimethoxyphenyl)methyl]-6,7-dimethoxyisoquinoline) belongs to the benzylisoquinoline-alkaloid class of compounds, with the isoquinoline being substituted at positions 6 and 7 by methoxy groups and at position 1 by a 3,4-dimethoxybenzyl group (Figure 1). It is a neutral solid with poor solubility in water. Papaverine is an antispasmodic drug which is used for the treatment of impotence and vasospasms (approved by the US Food and Drug Administration and non-FDA-approved) [5,6,7]. As a soft muscle relaxant, vasodilator and narcotic agent, papaverine has a direct relaxant action on the smooth muscle which may be attributed to its ability to inhibit phosphodiesterases and calcium channels [8,9,10]. It relaxes the smooth musculature of the larger blood vessels, particularly coronary, systemic peripheral and pulmonary arteries [11]. Papaverine increases cerebral blood flow and reduces cerebral vascular resistance through its immediate vasodilating action on cerebral blood vessels and lack of impact on oxygen intake [5,6,7]. Like quinidine, papaverine operates directly on the heart muscle to reduce conduction and extend the refractory period [12]. Papaverine also showed a potential antiviral activity against respiratory syncytial virus, cytomegalovirus, measles and HIV [13]. The biological half-life of papaverine hydrochloride given by the oral route is reported to be within the range of 1–2 h. Papaverine hydrochloride is rapidly absorbed orally and undergoes massive initial pass metabolism in the gut wall and liver, and the bioavailability is as low as 30% [10].

As a potential antitumor drug, papaverine showed selective and potential antitumor activity against several types of cancer cells, including breast carcinoma T47D [14], MCF-7 and MDA-MB-231 [15], prostate carcinoma PC-3 [16], LNCaP [17,18], colorectal carcinoma HT29 [14], hepatocarcinoma HepG2 [19] and fibrosarcoma HT1080 [14]. Papaverine was also found to sensitize A549 lung and EO771 breast tumor cells to radiation therapy by inhibiting the mitochondrial complex 1 [20]. Recently, papaverine has been identified—by in silico screening from the Drug Bank library—as a potential inhibitor for the receptor for advanced glycation end-products (RAGE). In this study, papaverine was found to dramatically inhibit HMGB/RAGE interaction and to suppress the production of inflammatory cytokines (IL-6 and TNF-α) [21]. In glioblastoma, papaverine significantly inhibited the cell proliferation of human temozolomide-sensitive U87MG and temozolomide-resistant T98G cells with EC_50_ values of 29 and 40 μM, respectively, by suppressing the interaction between HMGB1 and RAGE. Moreover, papaverine dramatically reduced tumor volume and delayed tumor growth in a human glioblastoma U87MG xenograft mouse model [22]. In a further study by the same group, the combination of papaverine with temozolomide significantly and more potently reduced the clonogenicity of T98G cells and delayed the tumor growth in a human glioblastoma U87MG xenograft mouse model [23]. Given these facts, papaverine has been considered as a potentially druggable scaffold for various tumor types and for RAGE inflammatory and immune disorders.

Metal ions play various crucial roles in human health, e.g., for their functions in drug’s action mechanisms and as indicative agents. In general, metals have several unique features, such as the power of redox activity, flexible coordination approaches and reactivity towards organic substrates [24]. Due to their reactivity, abnormal metal ion concentrations are related to different pathological disorders, including malignant growth [25]. Accordingly, metal ion complexes, either as medications or pro-drugs, are appealing for medicinal chemistry. Owning to their versatile reactivity, structure and geometry, metallodrugs provide a unique mode of action. Several studies indicated that the drug (or ligand) bioactivity improves upon binding to metal ions, which might be owed to the release of at least two biologically active species [26,27,28,29,30,31,32]. To investigate such a concept for a drug, one approach would be to examine the in vitro activity of the metal complexes including the drug as a ligand. The best examples include the conjugate of ferrocene with quinoline, which has completed the phase II clinical trials [30,33]; the octahedral polypyridyl meatal-complexes, which enhanced the levels of reactive oxygen species by targeting the mitochondria, and are used photodynamic therapy as photosensitizers [34,35,36,37,38,39,40]; and the nonsteroidal anti-inflammatory drugs coordinated with metals [28]. Among different metal complexes, ruthenium, gold and vanadium complexes have attracted particular attention. While quinoline Ru-complexes show potential antimicrobial activities [27,29], the *p*-cymene Ru-complexes show potential anticancer activities [41,42,43,44,45]. The vanadium-based complexes have been extensively investigated as potential anti-diabetic, anti-cancer, antibacterial, antiviral, anti-atherosclerotic and anti-tuberculosis drugs [46,47,48,49]. After the FDA’s approval of auranofin (tetra-*O*-acetylglucose-1-thiolgold(I) triethylphosphine complex) as a therapy for rheumatoid arthritis, the gold-based drugs have attracted special attentioin. The exploitation of gold complexes has led to vast diversity of gold compounds of biological relevance, including anti-cancer, anti-inflammatory and antiparasitic agents [50,51,52,53,54,55,56,57,58].

Although various structural and activity studies have been successfully performed for many drugs, papaverine–metal activity studies are very rare [59,60]. Indeed, only one entry of a crystal structure with papaverine as a ligand can be found in the Cambridge structural database [61]. These facts encouraged us to study the effects of complexing the bioactive benzylisoquinoline moiety of papaverine drug with a set of metals (V^+3^, Ru^+3^ and Au^+3^). Herein, we report the syntheses and structural characterizations of a novel set of papaverine–metal complexes. The biological activities, including the antibacterial and antitumor activities, of the papaverine and its metal derivatives, were evaluated using various microorganisms and human cancer cell lines (MCF-7 and Hep.G2).

## 2. Materials and Methods

### 2.1. General Description of Materials

All chemical reagents and solvents were purchased from Merck Co and used without further purification, unless otherwise specified. All the solvents were used after distillation by standard methods.

### 2.2. Instrumentation

Elemental analyses (carbon, hydrogen and nitrogen content) were verified using a Perkin–Elmer CHN 2400 in the Micro-analytical unit at the Faculty of Science, Cairo University, Egypt. The metal ions were determined gravimetrically by transforming the metals into their corresponding oxides. Molar conductivities of freshly prepared 1.0 mmol/dm^−3^ solutions in DMSO were assessed using Jenway 4010 conductivity meter. The UV–Vis spectra for papaverine and its metal complexes were determined for a solution of 1.0 mM in DMSO using UV2 Unicam UV/Vis Spectrophotometer with a 1 cm quartz cell. Magnetic measurements were performed on a Sherwood scientific magnetic balance using Guoy’s method and Hg[Co(CNS)_4_] as calibrants in the micro analytical laboratory, Faculty of Science, Mansoura University, Egypt. The infrared spectra of papaverine ligand and their metal complexes were recorded on Bruker FTIR Spectrophotometer (4000–400 cm^−1^) in KBr pellets. ^1^H and ^13^C-NMR spectra of papaverine ligand and metal complexes were recorded on a Varian Gemini 200 MHz spectrometer using DMSO-d_6_ as the solvent and TMS as an internal reference. Thermogravimetric analysis (TGA and DTG) was conducted in dynamic nitrogen atmosphere (30 mL/min) with a heating rate of 10 °C/min using a Schimadzu TGA–50H thermal analyzer. The X–ray powder diffraction experiments were carried out using a Rikagu diffractometer. The crystal surface was examined by employing scanning electron microscopy (SEM) using JEOL JSM–840.

### 2.3. Synthesis of Metal Complexes

A solution of MCl_3_ (0.1 mole, MCl_3_ = VCl_3_ (unhydrated), RuCl_3_.3H_2_O or AuCl_3_.3H_2_O) in methanol (0.5 M) was treated under stirring with a methanolic solution of papaverine hydrochloride (0.1 mol, 0.5 M). The pH of resulting mixture was then adjusted to pH ≈ 8 by carefully addition of methanolic ammonia solution (0.1 mol). After the resulting reaction mixture was allowed to stir under the same conditions for additional 3–4 h (during while a solid was formed), the mixture was filtered. The obtained solid product was washed two times with methanol and dried to afford the corresponding papaverine metal complexes in yields of 75–78% (details in Table 1). The obtained solids were re-crystallized to afford pure products, as indicated by analytical analysis. The metal complexes were used directly without any further purification steps.

Au(III)–papaverine complex: Yield 77%. ^1^H NMR (DMSO-d_6_, 400 MHz, ppm): δ 8.25 (d, 1H, arm.3), 7.52 (d, 1H, arm. 4), 7.30 (s, 1H, arm.8), 6.81 (s, 1H, arm. 2′), 6.79 (d, 1H, arm. 5), 6.76 (s, 1H, arm 6′), 6.76 (s, 1H, arm 5′), 4.47 (s, 1H, -CH_2_-), 3.65 (s, 3H, OCH_3_), 3.68 (s, 3H, OCH_3_), 3.86 (s, 3H, OCH_3_), 3.93 (s, 3H, OCH_3_). ^13^C NMR (DMSO-d_6_, 120 MHz, ppm): δ 158.3, 152.6, 150.0, 149.0, 147.5, 141.0, 133.3, 132.7, 122.5, 41.2, 118.9, 121.0, 118.9, 112.4, 106.1, 104.7, 56.2, 56.1, 55.9, 55.9.

### 2.4. Magnetic Susceptibility Measurements

Magnetic susceptibility measurements were carried out on a Sherwood Scientific magnetic balance according to Guoy’s method. The calculation was carried out using following equation:

X_g_ = C (R − R_0_)/10^−9^ M

where X_g_ is mass susceptibility per gram of sample, C is the calibration constant of the instrument and equal to 1.135, R is the balance reading for the sample and tube, R_0_ is the balance reading for the empty tube, M is the weight of the sample in grams and T is the absolute temperature.

### 2.5. Antibacterial Investigation

The antibacterial activities of papaverine and its metal complexes were tested against the gram-negative bacteria *Klebsiella pneumonia* and *Escherichia coli*, and the gram-positive bacteria *Staphylococcus epidermidis* and *Staphylococcus aureus*. The agar hole-well diffusion technique with diameter 4 mm was applied [62]. The investigated isolates of bacteria were cultivated in tubes and supplemented with nutrient broth. The seeded nutrient broth (1 cm^3^) was homogenized in the tube with 9 cm^3^ of melted nutrient agar (45 °C). The homogeneous suspension was filled into Petri dishes, and the holes were made in the cool medium. After cooling, 2 × 10^−3^ dm^3^ of papaverine or one of its metal complexes (at concentration of 1.0 mmol/dm^3^) was applied in these holes. The dishes were incubated at 25–27 °C for 24 h, and then the inhibition zone diameters were measured and expressed in mm. The antibacterial activities of examined probes were compared to the activities of augmentin and unasyn at the same concentrations.

### 2.6. Anticancer Investigation

The two cell lines, MCF-7 and HepG-2, were cultured in Dulbecco’s modified Eagle’s medium (DMEM) supplemented with 10% heat-inactivated fetal bovine serum, 1% *L*-glutamine, HEPES buffer and 50 µg/mL gentamycin. All cell lines were incubated at 37 °C under a humidified atmosphere of 95% air and 5% CO_2_ and were sub-cultured two times/week. All stock solutions were prepared in DMSO and the final concentration of DMSO in medium did not exceed 1% (*v*/*v*), at which cell viability was not inhibited. After the cells were allowed to resume exponential growth for 24 h, they were exposed to drugs at different concentrations in media for 72 h. The antitumor activity levels of papaverine and the corresponding Au complex were evaluated in vitro for comparisons with cisplatin and doxorubicin drugs using the viability assay [63]. The 50% inhibitory concentration (IC_50_), the concentration required to cause toxic effects of 50% in the intact cells, was estimated from graphic plots of the dose–response curves using Graphpad Prism software (San Diego, CA, USA). Evaluation was based on means from at least three independent experiments.

## 3. Results and Discussion

### 3.1. Elemental Analysis and Molar Conductivities of Papaverine Metal Complexes

The results of the elemental analysis and some physical properties of papaverine metal complexes are given in Table 1. The prepared complexes were colored and air-stable with a high melting point (300 °C). They were not water-soluble complexes but soluble only in DMF or DMSO. The chloride content in all prepared complexes was determined potentiometrically by the titration against a standard solution of AgNO_3_ which showed that none of the prepared complexes contained ionic chloride [64]. The molar conductivity values for the papaverine–metal complexes (1.0 mmol) in DMSO were in the range of 17.0–35 Ω^−1^ cm^−1^ mol^−1^. The Au(III) complex showed 17 Ω^−1^ cm^2^ mol^−1^, while Ru(III) and V(III) complexes showed 35 and 32 Ω^−1^ cm^2^ mol^−1^, respectively. Conductivity measurements provide information about the degree of ionization of the complex and the mode of chelation of the metal (i.e., the geometry of metal complex). The more molecular ions that a complex liberates in solution, the higher the molar conductivity value [65,66]. Although the V(III) and Ru(III) complexes showed almost duplicate conductivity values, all the three complexes were non-electrolytic. These results were also supported by the negative test for the chloride ions for all complexes.

### 3.2. Magnetic Susceptibility Measurements

Magnetic susceptibility measurements were carried out on a Sherwood Scientific magnetic balance according to Guoy’s method [67]. The calculation was carried out using following equations:

*X*_g_ = *C* (R − R_0_)/10^−9^ M

*X*_m_ = *X*_g_ * MWt

$$\mu_{\mathrm{eff}}\;=\;2.828\;{\sqrt{X}}_{m}*T$$

where *X*_g_ is mass susceptibility per gram of sample, *C* is the calibration constant of the instrument (*C* = 1.135), R is the balance reading for the sample and tube, R_0_ is the balance reading for the empty tube, M is the weight of the sample in grams and T is the absolute temperature. The V(III) complex showed a magnetic moment of 1.91 B.M. with expected hybridization of d^2^sp^3^, while the Ru(III) and Au(III) complexes showed magnetic moment values of 2.05 and 0.63 B.M., respectively, with dsp^2^ expected hybridization. These results indicated that all metal complexes have an octahedral geometry [68].

### 3.3. UV–Vis Spectra

The ultraviolet–visible electronic spectra of the papaverine HCl ligand and its metal complexes in DMSO solution were assigned (Figure S1). The papaverine ligand showed one sharp band at 316 and 334 nm attributed to π→π* and n→π* electronic transitions, respectively. For all complexes it showed the blue shifted electronic transition at 325 nm and it also showed the electronic transitions of the metal d orbitals (d-d electronic transition) observed located in the visible region (400–700 nm) as extra information under complexation [69].

### 3.4. IR Spectral Studies

The IR spectra of papaverine hydrochloride and its metal complexes are shown in Figures S2–S5 (see Supporting Information) and band assignments are given in Table 2. In all three complexes, the υ(C=N) frequency either shifted towards higher values or toward lower values, indicating involvement of nitrogen in coordination with metal ions [70,71].

Notably, the intensity of the υ(C=N) peak decreased in all metal complexes, which also indicates the involvement of the nitrogen atom of the iso-quinone moiety in the metal coordination. The rest of the IR peaks have almost the same values, indicating non-participation with metal ions [72,73,74]. The coordination of the metal ions via the nitrogen of the ligand was confirmed by the presence of peaks at 408–467 and 364–372 cm^−1^ due to υ(M–N) and υ(M–Cl) assignments.

### 3.5. ^1^H and ^13^C-NMR Spectral Studies

The ^1^H-NMR spectrum of the Au(III)–papaverine complex provided evidence for the mode of coordination (Figures S6 and S7). In the ^1^H-NMR spectrum of the papaverine ligand, the distinguished singlet peaks of 3′-OCH_3_, 4′-OCH_3_, 6′-OCH_3_ and 7′-OCH_3_ groups (at 3.58, 3.59, 3.74 and 3.70 ppm, respectively) were also present in the spectra of the Au(III) complex without any shift, indicating the absence of complexation of the oxygen atom of methoxy groups with the Au(III) ion. The ^1^H-NMR spectrum of Au(III) complex exhibited some similarities to the papaverine ligand with the occurrence of –CH_2_– (1a) and aromatic protons signals centered at 4.472 ppm and in the range of 6.761–8.254 ppm, respectively (Table 3) [75].

From the ^13^C-NMR spectrum, the Au(III) complex also exhibited some similarities to the ligand with the occurrence of carbon signals for OCH_3_ groups, -CH_2_- (1a), and aromatic rings (55.87, 55.93, 56.11 and 56.16 ppm) centering at 41.23 ppm and 104.75–158.35 ppm, respectively. Moreover, the aromatic carbon signals of the complex and ligand were located at the downfield region in the range of 104.75–158.35 ppm (Table 4). In general, there is no un-characterized peak in the ^1^H and ^13^C-NMR spectra of ligand and Au(III) complex, which indicates the purity of the product obtained.

### 3.6. Thermal Analysis

The thermogravimetric analyses (TGA) for all synthesized complexes are shown in Figures S8–S10 (see Supporting Information). The TGA indicated that all metal complexes are decomposed in a three stages process.

#### 3.6.1. V(III) Complex

This complex was thermally decomposed in three steps as follows: 180–260 °C, 290–390 °C and 410–550 °C, respectively (Figure S8). In the first step, the V(III) complex lost two molecules of ammonia. In the second step, the complex lost three chloride ions in the form of hydrochloride; and lastly, the complex lost the entire organic moiety. These results were in agreement with the theoretical weight loss and observed weight loss for each step of degradation (Table 5).

#### 3.6.2. Ru(III) Complex

As shown in Figure S9, this complex was thermally decomposed in three steps within the temperature range of 170–450 °C. The first step occurred in the range of 170–250 °C and involved the decomposition and the liberation of two ammonia molecules. Then, three chloride ions were removed in the form of hydrochloride within the temperature range 260–350 °C. The rest of the organic moiety was removed in the third step within the temperature range 360–450 °C. These results were in agreement with the theoretical weight loss and observed weight loss for each step of degradation (Table 5).

#### 3.6.3. Au(III) Complex

The TGA cure of this complex indicated that the mass loss started at 190 °C and continued up to 550 °C (Figure S10). The first weight loss corresponds to the liberation of two ammonia molecules that occurred in the range 190–280 °C. The second thermal decomposition step involved the loss of chloride ions as hydrochloride and took place within the temperature range of 300–380 °C. Continuous decomposition of the rest of the ligand molecule occurred in the third step within the temperature range 390–550 °C. The theoretical mass loss was in agreement with the observed weight loss for each step of degradation (Table 5).

### 3.7. X-Ray Powder Diffraction Studies

Any X-ray powder diffraction (XRD) pattern relies on the fact that each crystalline solid represents a definite compound of a definite structure and pattern. XRD patterns in the 4° < 2θ < 70° of the metal complexes were acquired to get the lattice dynamics for the metal complexes (Figure S11). The structural identification of the metal complexes was done following previously reported methods [74,75,76,77]. The XRD patterns of papaverine metal complexes under the present study revealed that all metal complexes are semi-crystalline with a nanoscale range at 127 nm for V(III)–papaverine complex, 135 nm for Ru(III)–papaverine complex and 124 nm for Au(III)–papaverine complex.

### 3.8. Scanning Electron Microscopy

**Figure 2:**
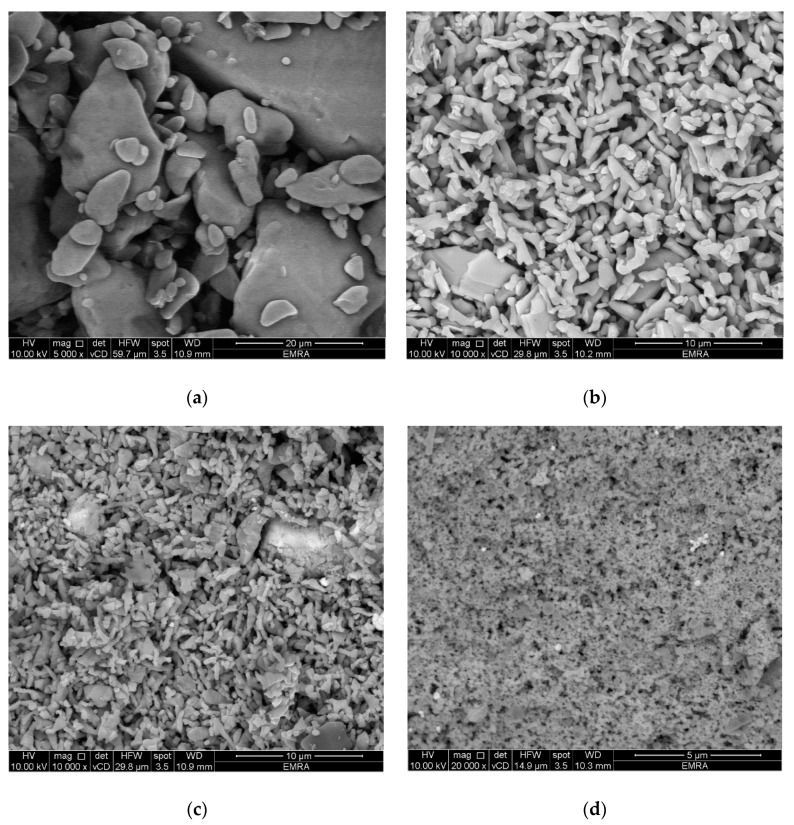
SEM of papaverine HCl (**a**) in complex with V(III) (**b**), Ru(III) (**c**) and Au(III) (**d**).

Scanning electron micrographs (SEM) for papaverine and its metal complexes were recorded and are shown in Figure 2. SEM provides high resolution, clear and less distorted images of the particles of the examined compounds, which helps in understanding the morphological changes upon metal complexation. SEM images of a metal complex’s beads show the changes in layers which are due to the interaction of the metal ion with the ligand and the adopted and well-defined geometry of the metal complex. As shown in Figure 2, SEM images of papaverine and its metal complexes under study showed a morphological difference with numerous territorial patches between them. The surfaces of the metal complexes consist of very small needle-like crystals that are in homogeneous forms. The particles of papaverine metal complexes have shapes and morphologies that differ from the parent papaverine ligand [77]. Crystals were found to grow up from just a single molecule to several molecules in an aggregate distribution with a particle size ranging from a few nanometers to over a hundred nanometers.

### 3.9. Proposed Structure of Papaverine Metal Complexes

**Figure 3:**
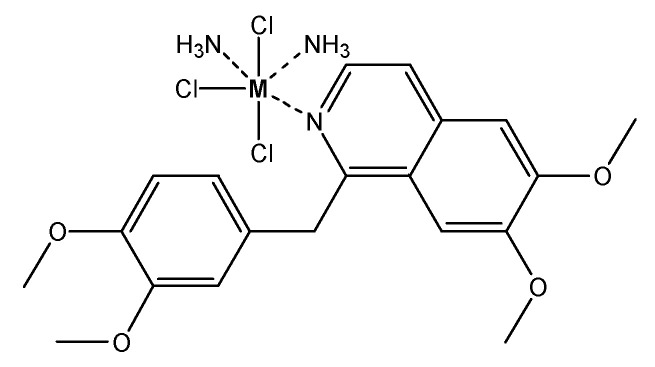
Proposed structure of M(III)–papaverine complex, where M = V(III), Ru(III) or Au(III).

As we have discussed before, we were not able to obtain single crystals for the papaverine metal complexes in order to perform X-ray studies. However, based on the results from the full analytical characterization of the prepared complexes (elemental analysis, magnetic properties, molar conductivity, thermogravimetric analysis and spectroscopic analysis), the papaverine ligand is coordinated to the metal(III) chloride as a neutral mono-dentate ligand through the lone pair of electrons of the nitrogen atom at the iso-quinoline moiety. Additionally, the results revealed an octahedral geometry around the metal center with two coordination sites with ammonia. The suggested structure of the papaverine metal complexes is shown in Figure 3.

### 3.10. Anti-Bacterial Study

The activities of the papaverine drug and its metal complexes against various microorganisms are presented in Table 6. The agar hole-well diffusion technique was applied, and the activities of compounds were evaluated at a concentration of 1.0 mmol/dm^3^. A clear zone around a disc indicated the inhibitory activity of a compound against an organism. The activity of tested compounds was evaluated in comparison to that of control (DMSO alone or augmentin). As detailed in Table 5, papaverine showed significant inhibition against almost all examined microorganisms. Several studies have shown that metal complexation significantly improves the antimicrobial activity of the parent ligand [15,19]. Unexpectedly, in contradiction to various studies, the activity of papaverine was dramatically decreased upon metal complexation. The Au(III) complex, among all complexes, was the most active metal complex against the examined microorganisms, while V(III) complex was the least active complex against the examined microorganisms.

### 3.11. Anticancer Study

Since the papaverine–Au(III) complex was the most active complex against microorganisms, we went forward to investigate further the antitumor activity for this complex (Figures S12 and S13). The anticancer activity of Au(III) complex was evaluated against two human cancer cell lines (MCF-7 and HepG-2) using a viability assay. The IC_50_ values for papaverine were 30.5 ± 1.1 and 58.5 ± 13.5 µg/mL against MCF-7 and HepG-2 cells, respectively (Table 7 and Table 8). Previous studies showed that papaverine inhibited cell proliferation in human glioblastoma U87MG and T98G cells with EC_50_ of 29 and 40 μM, respectively [21]. Interestingly, the anticancer activity of the papaverine drug was significantly improved upon complexation with gold(III) ions. The Au(III) complex showed IC_50_ values of 2.87 ± 0.12 and 21.6 ± 8.9 µg/mL against MCF-7 cells and HepG-2 cells, respectively. Similarly, the IC_50_ values of the standard anticancer drugs cisplatin and doxorubicin were 5.71 ± 0.5 and 0.35 ± 0.03 µg/mL, respectively, against MCF-7 cells, and 3.67 ± 0.2 and 0.36 ± 0.04 µg/mL, respectively, against HepG-2 cells. Noteworthily, the antitumor activity of Au(III) complex was higher than that of anticancer drug cisplatin. In order to investigate the biocompatibility of the Au–papaverine complex, we have examined the cytotoxicity of the Au–papaverine complex and the papaverine drug in normal cell lines (Table S1). The results showed that the Au complex is less toxic than papaverine alone with IC_50_ ≈111µg/mL. These results clearly indicate that the papaverine–Au(III) complex is a highly effective and selective anticancer drugs in both human MCF-7 and HepG-2 cells. Several studies showed that papaverine is a potential inhibitor of proliferation in various cancer cells and solid tumors, such as prostate, colorectal, breast and hepatocarcinoma ones [15,19,20,77]. Our novel findings indicate that the Au(III) complex of papaverine is a potential anticancer drug. Further studies should be done in future to investigate and confirm the in vivo anticancer activity of Au(III) complex.

## 4. Conclusions

In this study, we described the synthesis of novel papaverine-metal (V^+3^, Ru^+3^ and Au^+3^) complexes. The structures of the synthesized complexes were characterized by elemental analysis, molar conductivity, TGA, SEM and several spectroscopic techniques (UV–Vis, XRD, SEM, NMR), which indicated octahedral geometry for these complexes. Biological evaluation of synthesized metal complexes revealed that the papaverine–Au(III) complex, among the complexes we synthesized, possesses potential anticancer activity against both breast cancer MCF-7 cells and human HepG-2 cells. The anticancer activity of Au complex against different cancer cell lines was higher than that of the papaverine ligand alone, which indicates that Au metal complexation improved the anticancer activity of the parent ligand. Interestingly, the Au–complex showed anticancer activity against MCF-7 (IC_50_ 2.87 µg/mL), better than that of cisplatin. Overall, these results indicate that the Au(III)–papaverin complex is a promising antitumor compound that would make it a suitable candidate for further in vivo investigations.

## Figures and Tables

**Table 1:** Elemental analysis and physical properties of papaverine metal complexes.

Complex	M.Wt	Yield (%)	mp/(°C)	Color	Content [Calculated (Found)]			
					% C	% H	% N	% M
C_20_H_27_Cl_3_N_3_O_4_V	530.75	75	300 °C	Brown	45.21	5.09	7.91	9.60
					(45.18)	(5.03)	(7.90)	(9.58)
C_20_H_27_Cl_3_N_3_O_4_Ru	580.88	78	300 °C	Dark brown	41.35	4.69	7.23	17.40
					(41.30)	(4.61)	(7.20)	(17.39)
C_20_H_27_Cl_3_N_3_O_4_Au	676.77	77	300 °C	Dark green	35.41	4.00	6.13	28.65
					(35.50)	(4.02)	(6.21)	(29.10)

**Table 2:** IR characteristics of papaverine hydrochloride and its complexes.

Frequency (cm^−1^)				Assignment
Papaverine	V(III)	Ru(III)	Au(III)	
3011	3018	3018	3011	CH (stretch); aromatic rings
29742938	2967	2967	2961	CH (asymmetric stretch); OCH_3_
2836	2836	2843	2836	CH (symmetric stretch); OCH_3_
1635	1618	1618	1611	C=N (stretch)
1517	1517	1509	1509	C=N (in conjugated cyclic system)
1458	1407	1466	1407	
1604	1590 1560	1560	1589 1560	C=C (aromatic)
-	467, 415	267, 415	467, 408	M–N (stretch)
	372	364	364	M–Cl (stretch)

**Table 3:** ^1^H-NMR characteristics of papaverine HCl and its Au(III) complex.

Atoms	δ_H_(ppm)	
	Papaverine HCl	Au(III) Complex
1a	4.35	4.47
3	8.18	8.25
4	7.21	7.52
5	6.80	6.79
8	7.14	7.30
2′	6.68	6.81
5′	6.57	6.76
6′	6.65	6.76
3′-OMe	3.58	3.65
4′-OMe	3.59	3.68
6′-OMe	3.74	3.86
7′-OMe	3.70	3.93

**Table 4:** ^13^C-NMR characteristics of papaverine HCl and its Au(III) complex.

Atoms	δ_C_(ppm)	
	Papaverine HCl	Au(III) Complex
1	157.7	158.3
1a	42.1	41.2
3	140.8	140.9
4	118.6	118.9
4a	133.3	133.3
5	105.2	104.7
6	152.3	152.6
7	149.7	150.0
8	104.1	106.1
8a	122.8	122.5
1′	132.2	132.7
2′	111.9	112.4
3′	147.5	147.5
4′	149.0	149.0
5′	112.0	118.9
6′	120.5	121.0
6-OMe	55.8	55.9
7-OMe	55.8	55.9
3′-OMe	55.8	56.1
4′-OMe	55.8	56.2

**Table 5:** TGA data for V(III), Ru(III) and Au(III)–papaverine complexes.

Complex	Decomp. Step	Temp. Rang	TG Weight Loss (%)		Assignments
			Calc.	Found	
V(III) complex	1	180–260 °C	6.41	6.00	2NH_3_
	2	290–390 °C	20.63	20.50	3HCl
	3	410–550 °C	57.28	57.20	C_20_H_18_NO_2_
Ru(III) complex	1	170–250 °C	5.85	6.00	2NH_3_
	2	260–350 °C	18.85	18.35	3HCl
	3	360–450 °C	52.33	52.00	C_20_H_18_NO_2_
Au(III) complex	1	190–280 °C	5.02	5.00	2NH_3_
	2	300–380 °C	16.18	16.10	3HCl
	3	390–550 °C	49.65	49.44	C_20_H_18_NO_4_

**Table 6:** Antibacterial activities (inhibition zone diameter, mm/µg sample) of papaverine and its metal complexes.

Ligand/Complex	*K. pneumonia*	*E. coli*	*S. epidermidis*	*S. aureus*
Control, DMSO	0.0	0.0	0.0	0.0
Papaverine	0.4	0.2	0.5	0.5
V(III) complex	0.0	0.1	0.3	0.0
Ru(III) complex	0.3	0.1	0.2	0.0
Au(III) complex	0.3	0.3	0.2	0.0
Augmentin	0.5	0.3	1.0	0.4
Unasyn	0.2	0.1	1.0	0.2

**Table 7:** The anticancer effects of papaverine HCl and its Au(III) complex in human MCF-7 cells.

Sample Conc. (µg/mL)	Papaverine HCl			Au(III) Complex			Cisplatin *			Doxorubicin *		
	Viability %	Inhibitory %	S.D. (±)	Viability %	Inhibitory %	S.D. (±)	Viability %	Inhibitory %	S.D. (±)	Viability %	Inhibitory %	S.D. (±)
500	6.23	93.77	0.21	3.16	96.84	0.25	3.72	96.28	0.12	1.51	98.49	0.17
250	13.91	86.09	0.13	5.38	94.62	0.14	4.98	95.02	0.24	2.36	97.64	0.26
125	20.42	79.58	0.43	10.21	89.79	0.07	7.83	92.17	0.61	3.21	96.79	0.21
62.5	32.76	67.24	0.92	14.59	85.41	0.57	14.68	85.32	0.23	5.07	94.93	0.32
31.25	48.97	51.03	1.41	21.63	78.37	0.29	23.79	76.21	0.41	6.93	93.07	0.29
15.6	71.25	28.75	2.53	27.96	72.04	0.18	34.62	65.38	0.89	15.46	84.54	1.07
7.8	87.43	12.57	0.41	35.82	64.18	0.24	46.71	53.29	1.37	19.89	80.11	1.27
3.9	97.82	2.18	0.06	46.29	53.71	0.95	52.85	47.15	0.98	24.98	75.02	1.30
2	100	0	0	52.86	47.14	0.78	61.74	38.26	0.36	31.69	68.31	0.82
1	100	0	0	65.03	34.97	0.13	70.88	29.12	0.16	40.17	59.83	1.53
0	100	0	0	100	0	0	100	0	0	100	0	0
IC_50_	30.5 ± 1.1 µg/mL			2.87 ± 0.12 µg/mL			5.71 ± 0.5 µg/mL			0.35 ± 0.03 µg/mL		

* Standard reference. All values are the averages at least of three independent experiments.

**Table 8:** The anticancer effects of papaverine HCl and its Au(III) complex in human HepG-2 cells.

Sample Conc. (µg/mL)	Papaverine HCl			Au(III) Complex			Cisplatin *			Doxorubicin *		
	Viability %	Inhibitory %	S.D. (±)	Viability %	Inhibitory %	S.D. (±)	Viability %	Inhibitory %	S.D. (±)	Viability %	Inhibitory %	S.D. (±)
500	8.85	91.15	0.13	5.83	94.17	0.15	3.08	96.92	0.04	1.72	98.28	0.42
250	20.72	79.28	0.46	11.74	88.26	0.28	4.31	95.69	0.17	2.70	97.30	0.50
125	28.94	71.06	0.59	20.36	79.64	0.14	6.75	93.25	0.21	4.22	95.78	0.36
62.5	46.75	53.25	2.31	29.40	70.6	0.26	12.39	87.61	0.18	6.13	93.87	0.39
31.25	72.31	27.69	3.45	38.64	61.36	1.82	22.98	77.02	0.41	13.05	86.95	0.72
15.6	88.42	11.58	1.06	57.03	42.97	2.35	31.87	68.13	0.91	18.13	81.87	1.16
7.8	95.17	4.83	0.35	73.19	26.81	2.48	40.62	59.38	0.86	20.81	79.19	1.22
3.9	99.63	0.37	0.11	87.28	12.72	1.54	47.89	52.11	0.67	25.59	74.41	0.89
2	100	0	0	96.31	3.69	0.53	60.75	39.25	1.83	29.50	70.50	0.75
1	100	0	0	99.76	0.24	0.12	68.17	31.83	0.54	38.39	61.61	1.05
0	100	0	0	100	0		100	0	0	100	0	0
IC_50_	58.5 ± 13.5 µg/mL			21.6 ± 8.9 µg/mL			3.67 ± 0.2 µg/mL			0.36 ± 0.04 µg/mL		

* Standard reference. All values are the averages at least of three independent experiments.

## Supplementary Materials

The following are available online, Figure S1: UV-Vis spectra of papaverine (ligand) (a) in complex with Au (III) (b), V (III) (c), and Ru (III) (d) after 0h (A) and 72h, (B). Figure S2: IR spectra of papaverine (ligand), Figure S3: IR spectra of V(III) –papaverine complex, Figure S4: IR spectra of Ru(III)-papaverine complex, Figure S5: IR spectra of Au(III) –papaverine complex, Figure S6: 1H,13C-NMR spectra for papaverine ligand, Figure S7: 1H,13C-NMR spectra for papaverine-Au (III) complex, Figure S8: TGA of V(III)-papaverine complex, Figure S9: TGA of Ru(III)-papaverine complex, Figure S10: TGA of Au(III)-papaverine complex, Figure S11: X-ray diffraction patterns of papaverine HCl (A), V(III)-papaverine complex (B), Ru(III)-papaverine complex (C), and Au(III)-papaverine complex (D), Figure S12: The anticancer effects of papaverine HCl and its Au(III) complex in human MCF-7 cells, Figure S13: The anticancer effects of papaverine HCl and its Au(III) complex in human HepG-2 cells. Table S1: Cell viability for papaverine and its Au(III) complex.
