# Supplementary material for: Novel Papaverine Metal Complexes with Potential Anticancer Activities
Source: Molecules. 2020 Nov 20;25(22):5447. doi: 10.3390/molecules25225447 (PMC7699950; doi:10.3390/molecules25225447)
Supplement: Supplementary file 1 [file molecules-25-05447-s001.pdf]

Supplementary Data

Novel Papaverine Metal Complexes with Potential Anticancer Activities

Ahmed Gaber [1], Walaa F. Alsanie [2], Deo Nandan Kumar [3], Moamen S. Refat [4,5,*] and Essa M. Saied [6,7,*]

1 Department of Biology, College of Science, Taif University, P.O. Box 11099, Taif 21944, Saudi Arabia; a.gaber@tu.edu.sa
2 Department of Clinical Laboratories, College of Applied Medical Sciences, P.O. Box 11099, Taif 21944, Saudi Arabia; w.alsanie@tu.edu.sa
3 Department of Chemistry, Deshbandhu College, University of Delhi, Delhi 110019, India; dnandan2002@gmail.com
4 Department of Chemistry, College of Science, Taif University, P.O. Box 11099, Taif 21944, Saudi Arabia
5 Department of Chemistry, Faculty of Science, Port Said University, Port Said 42511, Egypt
6 Chemistry Department, Faculty of Science, Suez Canal University, Ismailia41522, Egypt
7 Institute for Chemistry, Humboldt Universität zu Berlin, Brook-Taylor-Str. 2, 12489 Berlin, Germany
* Correspondence: moamen@tu.edu.sa (M.S.R.); saiedess@hu-berlin.de (E.M.S.)

Academic Editor: Kogularamanan Suntharalingam
Received: 20 October 2020; Accepted: 17 November 2020; Published: 20 November 2020

I- UV-Vis spectra

A

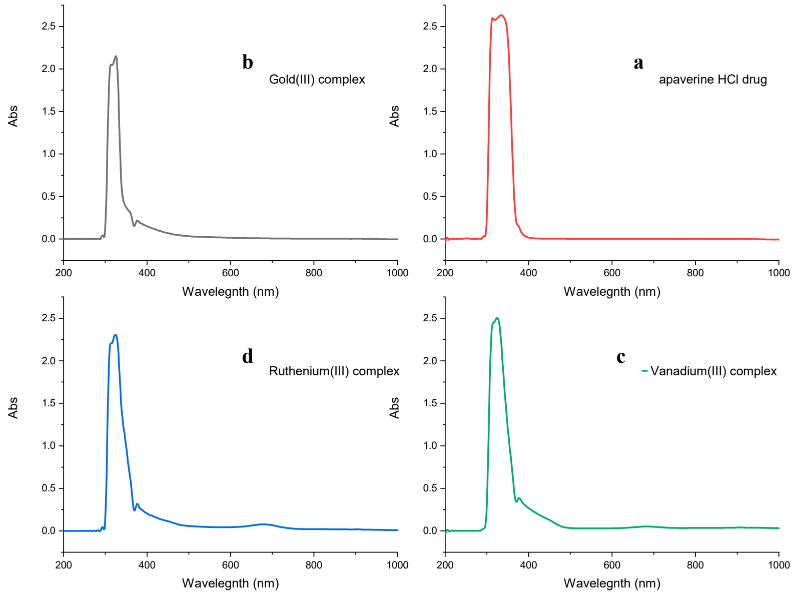

B

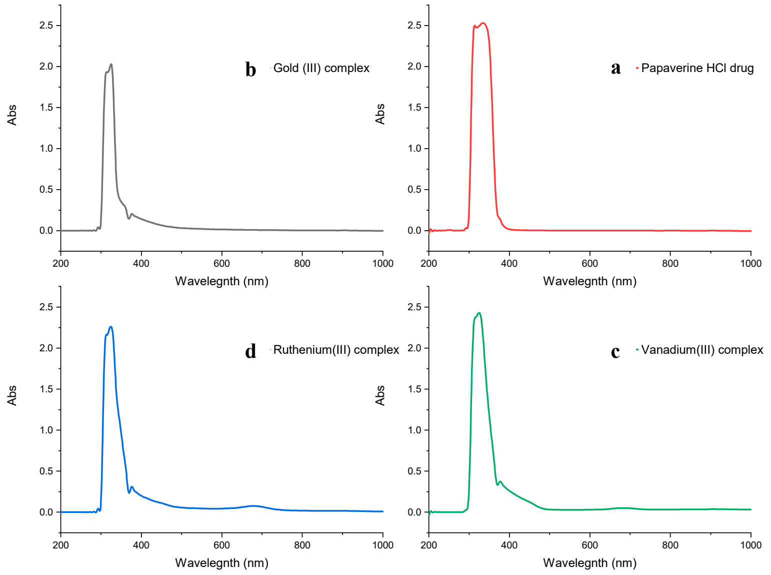

Figure S1. UV-Vis spectra of papaverine (ligand) (a) in complex with Au (III) (b), V (III) (c), and Ru (III) (d) after 0h (A) and 72h (B).

I- IR spectral for papaverine and synthesized complexes

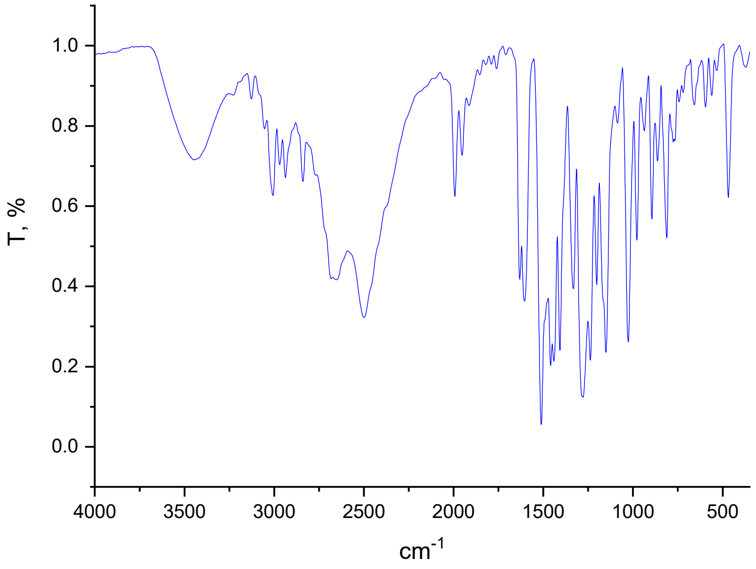

Figure S2. IR spectra of papaverine (ligand).

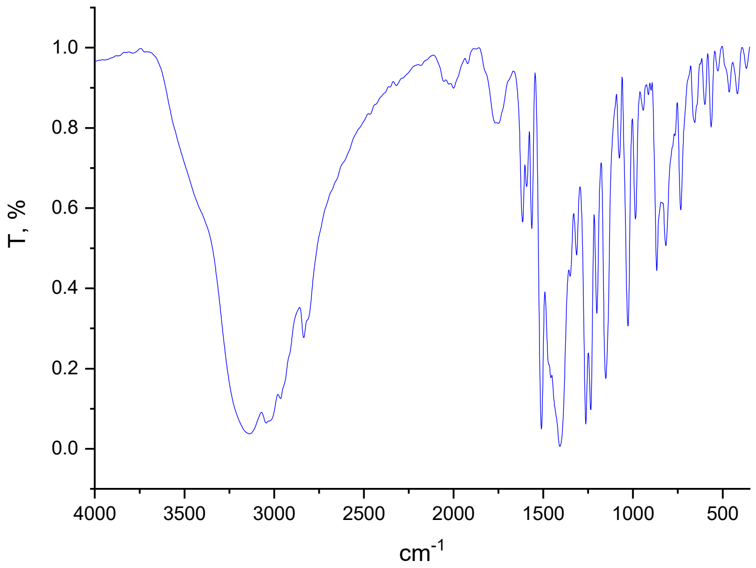

Figure S3. IR spectra of V(III) –papaverine complex.

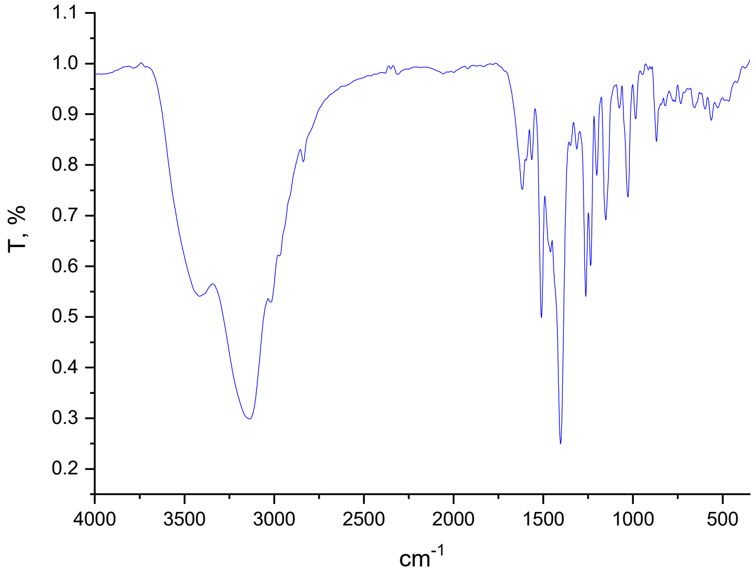

Figure S4. IR spectra of Ru(III)-papaverine complex.

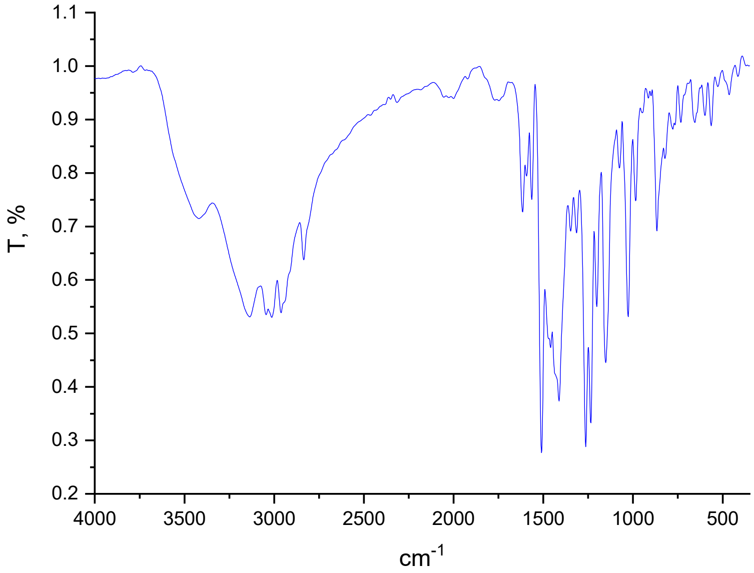

Figure S5. IR spectra of Au(III) –papaverine complex.

I- ^{1}H,^{13}C-NMR spectral studies

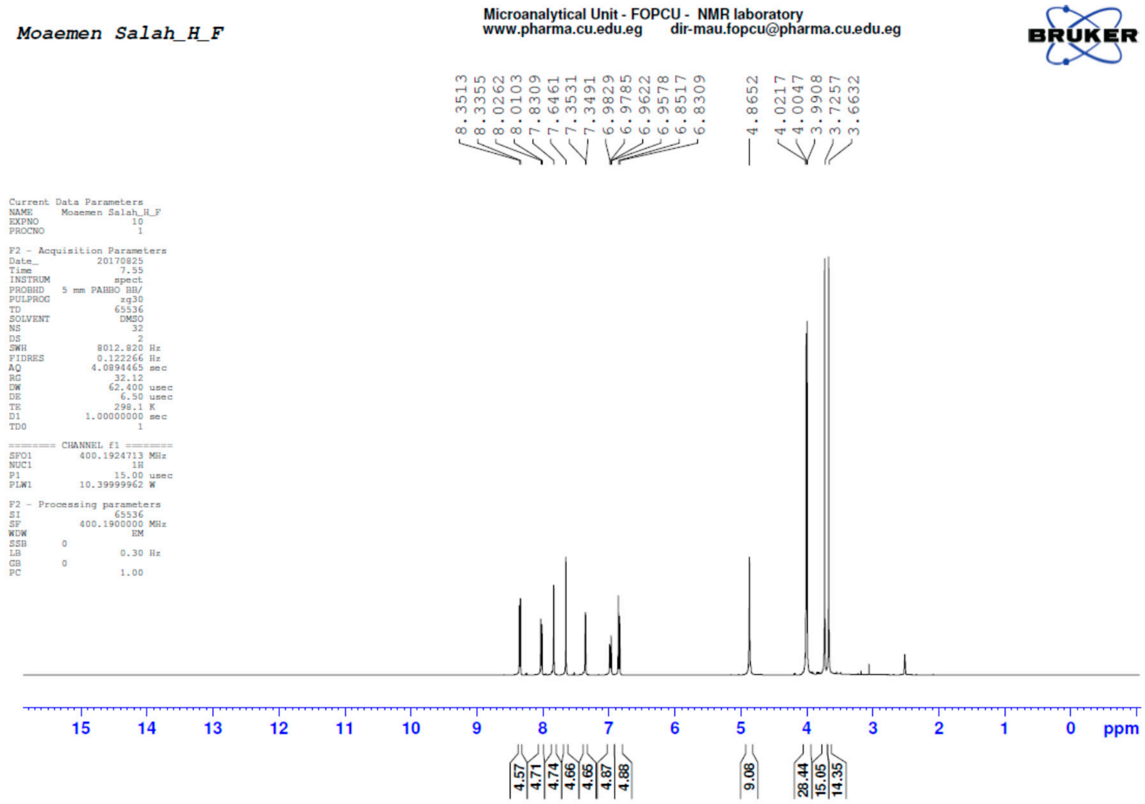

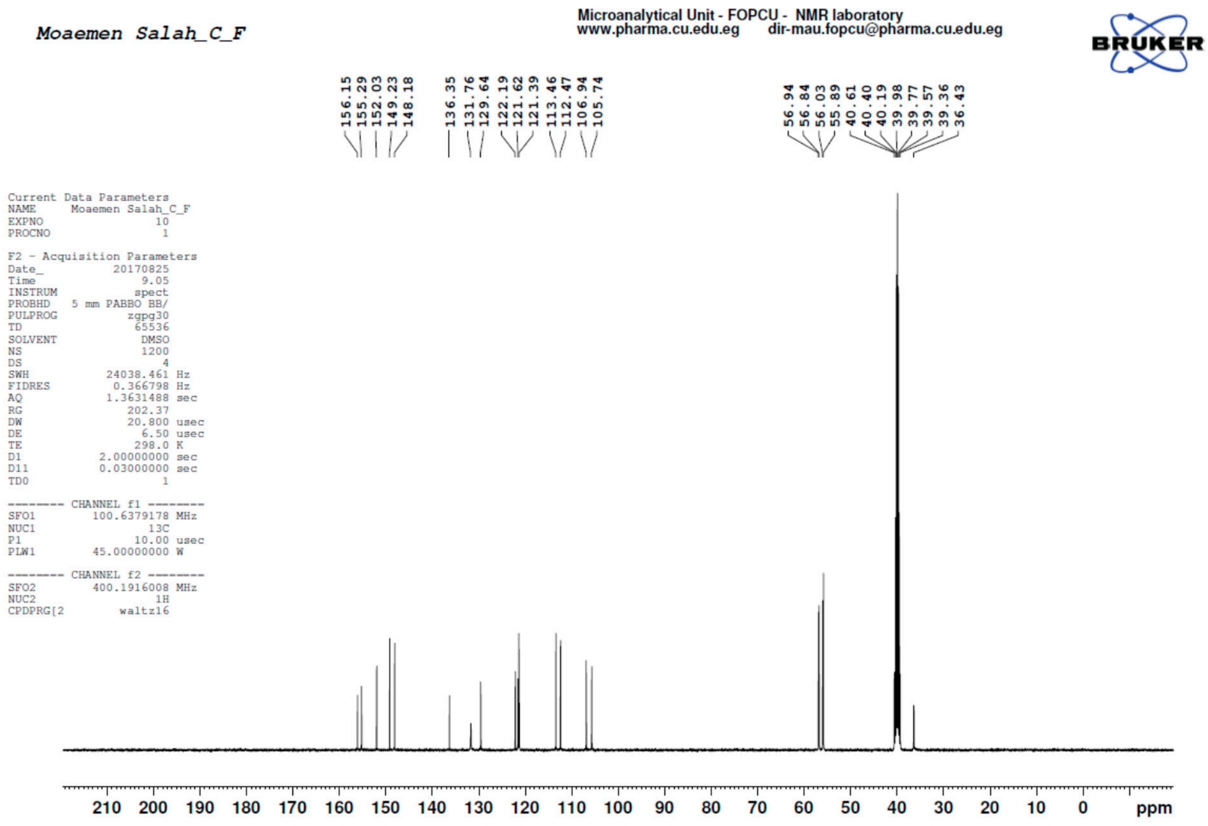

Figure S6. ^{1}H,^{13}C-NMR spectra for papaverine ligand.

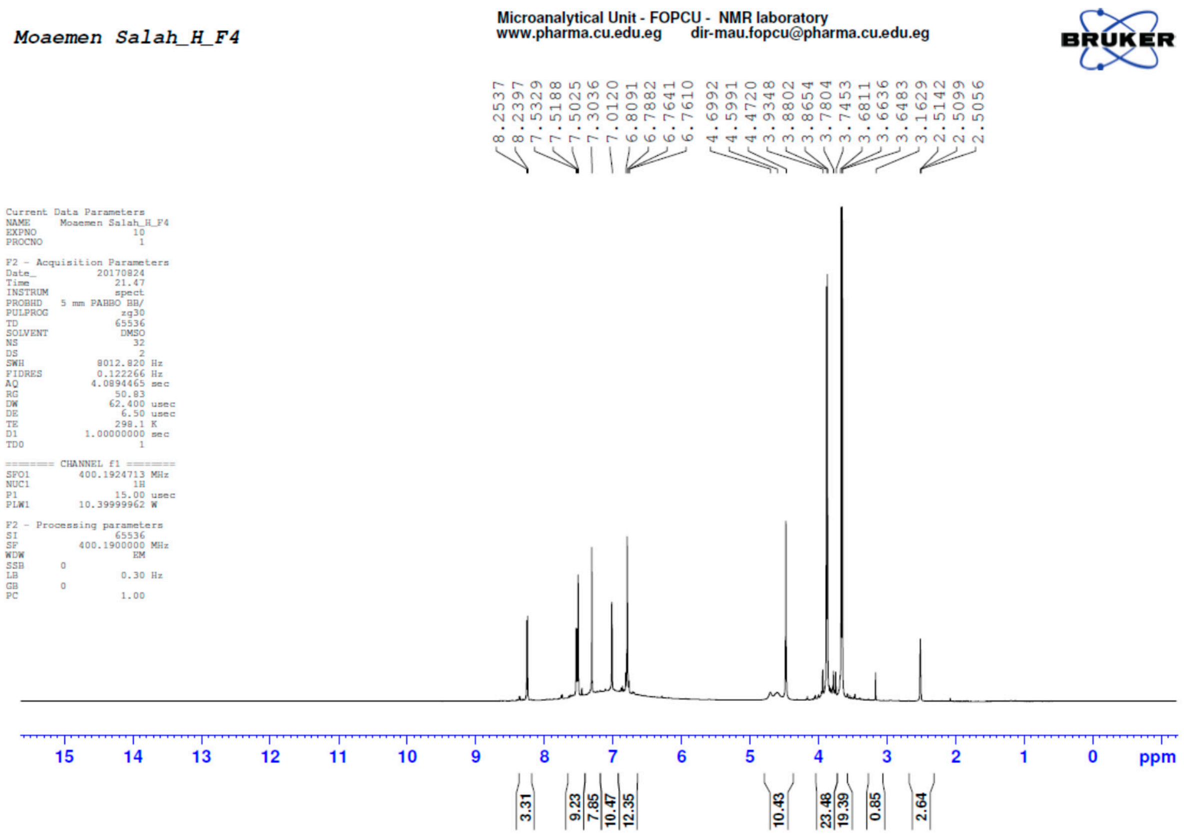

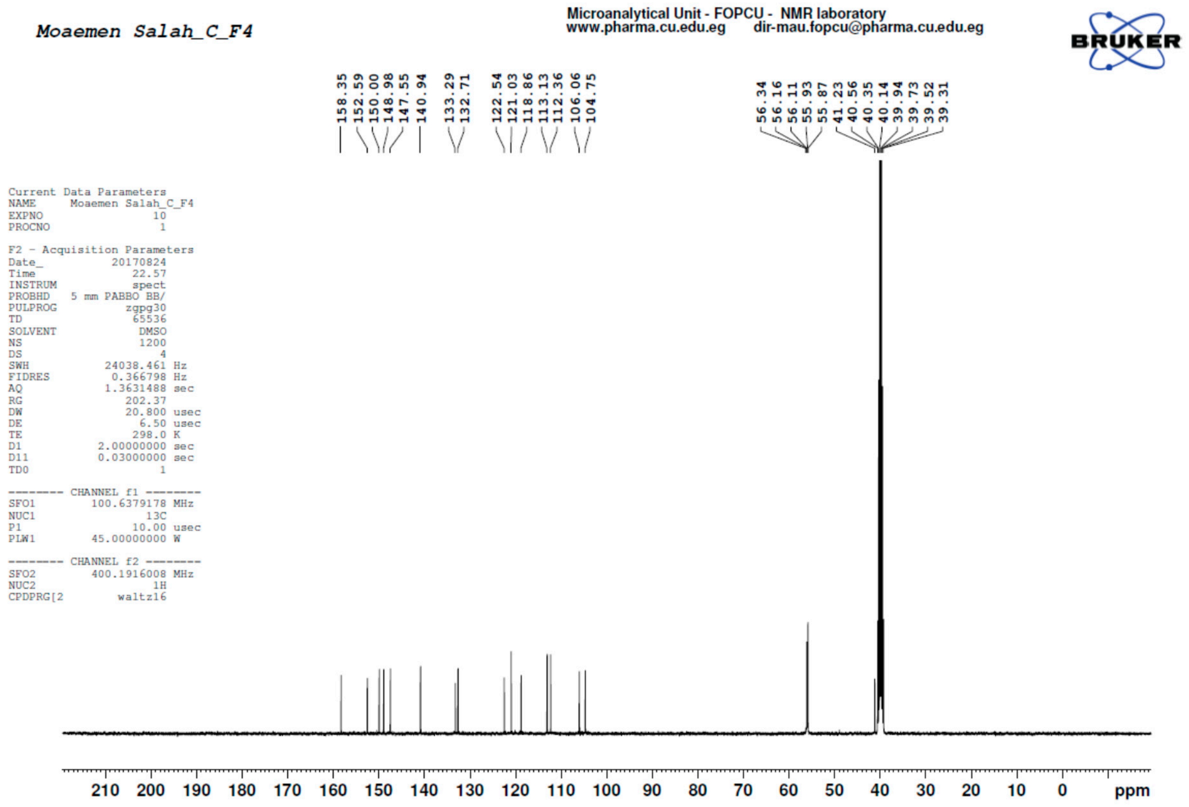

Figure 7. ^{1}H,^{13}C-NMR spectra for papaverine-Au (III) complex.

I- The thermogravimetric analysis (TGA) for all synthesized complexes

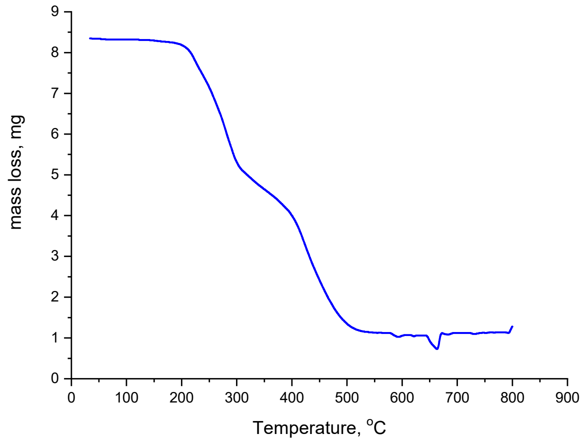

Figure S8. TGA of V(III)-papaverine complex.

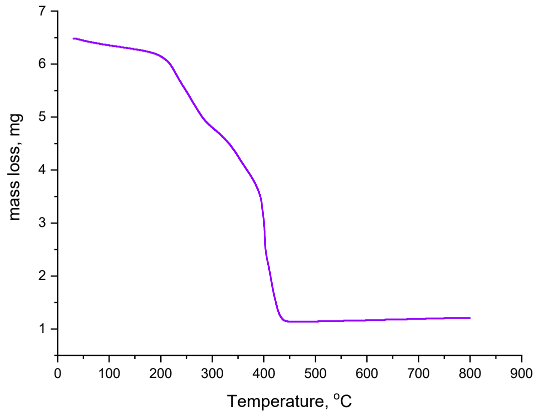

Figure S9. TGA of Ru(III)-papaverine complex.

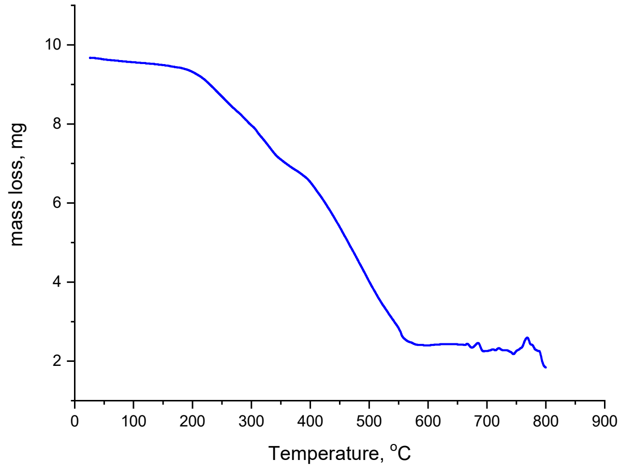

Figure S10. TGA of Au(III)-papaverine complex.

I- X–ray Powder diffraction

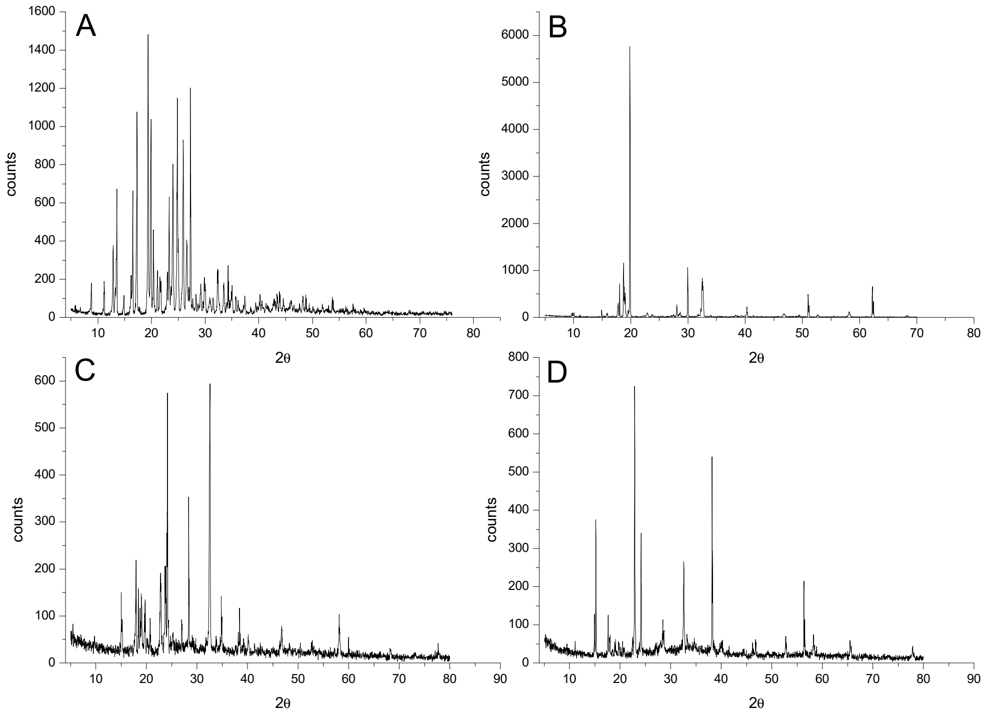

Figure S11. X-ray diffraction patterns of papaverine HCl (A), V(III)-papaverine complex (B), Ru(III)-papaverine complex (C), and Au(III)-papaverine complex (D).

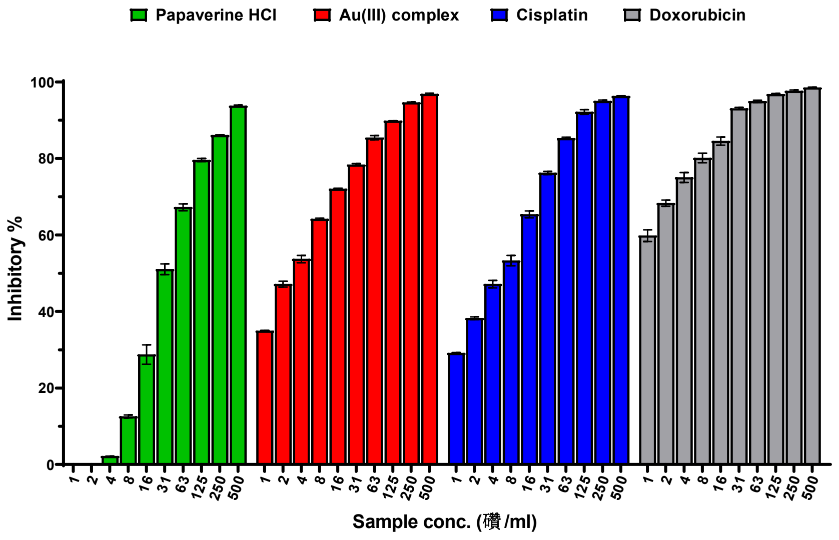

Figure S12. The anticancer effects of papaverine HCl and its Au(III) complex in human MCF-7 cells.

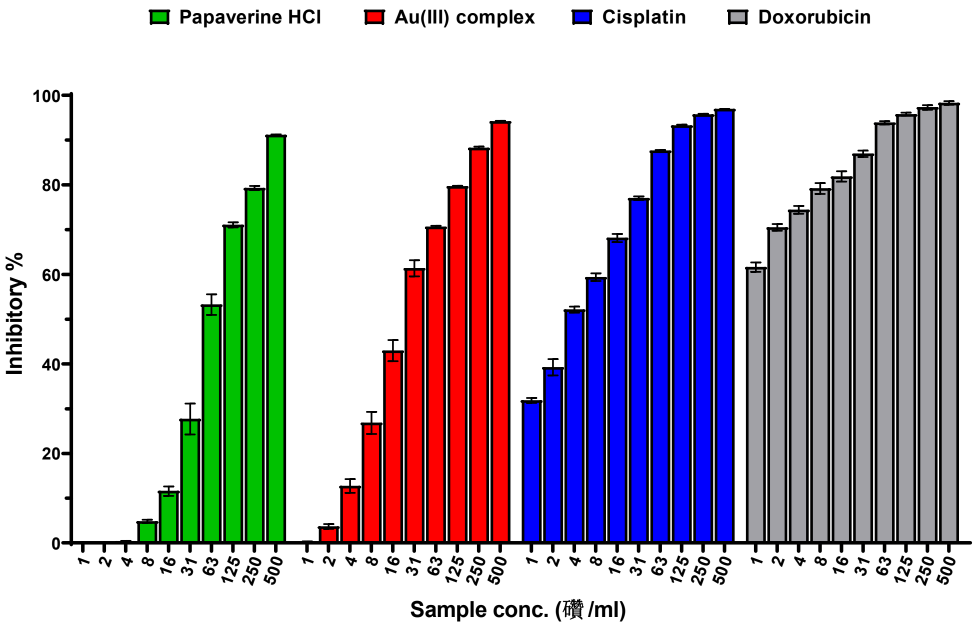

Figure S13. The anticancer effects of papaverine HCl and its Au(III) complex in human HepG-2 cells.

Determination of the biocompatibility in normal cell line (wi38 cells):

1-the 96 well tissue culture plate was inoculated with 1 X 10^5 cells / ml (100 ul / well) and incubated at 37°C for 24 hours to develop a complete monolayer sheet.

2- Growth medium was decanted from 96 well micro titer plates after confluent sheet of cells were formed, cell monolayer was washed twice with wash media.

3- two-fold dilutions of tested sample was made in RPMI medium with 2% serum (maintenance medium).

4- 0.1 ml of each dilution was tested in different wells leaving 3 wells as control, receiving only maintenance medium.

5- Plate was incubated at 37°C and examined. Cells were checked for any physical signs of toxicity, e.g. partial or complete loss of the monolayer, rounding, shrinkage, or cell granulation.

6- MTT solution was prepared (5mg/ml in PBS) (BIO BASIC CANADA INC).

8- 20ul MTT solution were added to each well. Place on a shaking table, 150rpm for 5 minutes, to thoroughly mix the MTT into the media.

9) Incubate (37C, 5% CO2) for 1-5 hours to allow the MTT to be metabolized.

10) Dump off the media. (dry plate on paper towels to remove residue if necessary.

11) Resuspend formazan (MTT metabolic product) in 200ul DMSO. Place on a shaking table, 150rpm for 5 minutes, to thoroughly mix the formazan into the solvent.

12) Read optical density at 560nm and subtract background at 620nm. Optical density should be directly correlated with cell quantity.

References

1. Slater, T. et al. (1963) Biochem. Biophys. Acta 77:383. van de Loosdrecht, A.A., et al. J. Immunol. Methods 174: 311-320, 1994.

2 Alley, M.C., et al. Cancer Res. 48: 589-601, 1988

Table S1: Cell viability for papaverine and its Au(III) complex.

ID	Conc. ug/ml	O.D			Mean O.D	ST.E	Viability %	Toxicity %	IC50
**Wi38**	0	0.362	0.347	0.368	0.359	0.00624	100	0	
**Au (III) complex**	500	0.032	0.041	0.055	0.042667	0.006692	11.9	88.1	111.59
	250	0.086	0.084	0.079	0.083	0.002082	23.1	76.9	
	125	0.152	0.136	0.144	0.144	0.004619	40.1	59.9	
	62.5	0.268	0.289	0.285	0.280667	0.006438	78.12	21.8	
	31.25	0.356	0.341	0.355	0.350667	0.004842	97.67	2.3	
	15.6	0.361	0.357	0.356	0.358	0.001528	99.73	0.28	
	7.8	0.368	0.352	0.355	0.358333	0.00491	99.82	0.18	
	3.9	0.353	0.368	0.356	0.359	0.004583	100	0	
**papaverine HCl**	500	0.025	0.019	0.02	0.021333	0.001856	5.9	94.1	28.08
	250	0.041	0.026	0.038	0.035	0.004583	9.74	90.26	
	125	0.086	0.095	0.077	0.086	0.005196	23.96	76.04	
	62.5	0.124	0.136	0.128	0.129333	0.003528	36.03	63.976	
	31.25	0.152	0.178	0.169	0.166333	0.007623	46.33	53.67	
	15.6	0.241	0.236	0.258	0.245	0.006658	68.26	31.75	
	7.8	0.301	0.326	0.318	0.315	0.007371	87.74	12.26	
	3.9	0.358	0.342	0.364	0.354667	0.006566	98.79	1.21	
